# Relationship Between Vitamin D Level and Platelet Parameters in Children With Viral Respiratory Infections

**DOI:** 10.3389/fped.2022.824959

**Published:** 2022-04-07

**Authors:** Gavriela Feketea, Vasiliki Vlacha, Raluca Maria Pop, Ioana Corina Bocsan, Luminita Aurelia Stanciu, Anca Dana Buzoianu, Mihnea Zdrenghea

**Affiliations:** ^1^Department of Haematology, “Iuliu Hatieganu” University of Medicine and Pharmacy, Cluj-Napoca, Romania; ^2^Department of Paediatrics, Amaliada Hospital, Amaliada, Greece; ^3^Department of Paediatrics, Karamandaneio Children's Hospital, Patras, Greece; ^4^Department of Early Years Learning and Care, University of Ioannina, Ioannina, Greece; ^5^Department of Pharmacology, Toxicology and Clinical Pharmacology, “Iuliu Hatieganu” University of Medicine and Pharmacy, Cluj-Napoca, Romania; ^6^National Heart and Lung Institute, Imperial College London, London, United Kingdom; ^7^Department of Hematology, “Ion Chiricuta” Oncology Institute, Cluj-Napoca, Romania

**Keywords:** vitamin D, platelets, MPV, influenza, respiratory infections, children

## Abstract

Apart from their classical roles, both platelets and vitamin D play important roles in inflammation and infectious diseases. This study evaluated the platelet response to viral respiratory tract infection in children aged 4–16 years, 32 with influenza, 27 with non-influenza viral infection tested by nasopharyngeal swab and 21 healthy children of the same age. Blood count, including platelet count (PLT), mean platelet volume (MPV) and other platelet indices, erythrocyte sedimentation rate (ESR), C-reactive protein (CRP) and vitamin D (vit D) levels were compared. The influenza group showed lower PLT and platelet mass (PLT^*^MPV), and the non-influenza group showed significantly lower MPV, which was correlated with the vit D levels, but not CRP or ESR, and the value vit D^*^MPV was significantly lower in this group. These results revealed that platelet activation in viral respiratory tract infections in children, as measured by MPV, is related to the vit D level, with differences between influenza and non-influenza infection.

**Conclusions:**

Viral respiratory tract infection in children can diminish the platelet size most likely by suppressing the platelet activation. This response is associated with low levels of vit D. Whether the vit D status is associated with the virus-platelet immune/inflammatory process needs further investigation.

## Introduction

Acute respiratory tract infections (RTIs) are the most common infections worldwide (1). In a study conducted in previously healthy children by Taylor et al., the most common cause of RTIs was respiratory viruses, mainly rhinoviruses (42.2%), followed by influenza virus (15.8%) (2). The clinical symptoms involve the upper and lower respiratory tract, ranging from mild to severe, with a diagnosis including bronchiolitis, bronchitis, pneumonia, etc.

The incidence of RTIs related to influenza virus is higher among children aged <3 years, reaching 179/1,000 (3). One study analyzing hospitalization in children contracting influenza, reported the rate to be higher among infants under 6 months, with 15% of them needing admission to a pediatric intensive care unit (PICU) (4).

The complete blood count (CBC) is the most frequently used laboratory test during an infection process (5). The changes in the white blood cell (WBC) in response to infection are most commonly analyzed (6), but the platelet response to infection is diverse, with regard to both platelet count (PLT) and other platelet parameters, notably the mean platelet volume (MPV).

The primary function of platelets is haemostatic, but recently, their role in inflammation and immunogenicity has also been evaluated. They show the ability to recruit leukocytes and release proinflammatory and anti-inflammatory factors (7). Several disorders are known to be associated with platelet activation, including acute lung injury (8), inflammatory bowel disease (IBD) (9), rheumatoid arthritis (RA) and sepsis (10). The role of platelets in viral infections has also been documented (11). Specifically, platelet interaction has been described with adenovirus (12), dengue (13, 14), hepatitis C (15), and Epstein-Barr virus (EBV) (16).

The main role of vitamin D (vit D) is in bone homeostasis, but in recent years its role in infections (17), inflammation and immune response (18) has been documented. Vit D acts as a stimulant of innate defense (19), and as an immunomodulator in adaptive immunity. Low levels of vit D are associated with a variety of viral infectious diseases (20, 21). In patients with HIV-1 with low levels of vit D, a decrease in inflammation was observed when vit D supplementation restored the levels to normal (22). Vit D deficiency increases the susceptibility to enveloped viral infections, including respiratory syncytial virus (RSV) (23). With respect to influenza, vit D has been shown to enhance the immunogenicity of influenza vaccine in elderly subjects (24, 25), although other studies failed to confirm this finding (26).

In this study we investigated the platelet changes during influenza and non-influenza RTIs in children, and the possible role of vit D in the process.

## Materials and Methods

The study population consisted of Caucasian children, aged 4–16 years presenting with symptoms of RTI in the emergency department (ED) of a regional hospital in Greece during a 6-month period (September 2019–February 2020). The exclusion criteria were comorbidities, need for hospital admission, vit D supplement during the preceding 3 months, a diagnosis of bacterial infection, and administration of medication other than antipyretics. A control group was selected from children of the same age attending the pediatric outpatient clinic of the same hospital for a well-child visit, during the same time period. Similar exclusion criteria were applied for the control subjects. Children with even minor infections on the day of examination were excluded. The study was approved by the hospital ethics committee. Written, informed consent was provided by parents'care givers of all the children in the study.

Testing for Influenza A and B by a nasopharyngeal swab was performed on each child on arrival in the ED. The demographic characteristics of each child, and the duration and height of fever and the presence of cough were documented. A sample of venous blood was drawn for analysis of (CBC), erythrocyte sedimentation rate (ESR), C-reactive protein (CRP) and the level of vit D. The CBC was performed in a Unicel DxH 600 Coulter Cellular Analysis System by Beckman Coulter. The blood samples for CBC were obtained by ethylenediaminetetraacetic acid (EDTA) and were analyzed within 1 h after collection in order to prevent platelet swelling.

The influenza viruses were identified by a rapid immunochromatographic test for the qualitative detection of influenza antigens in nasopharyngeal swab. Vit D (25(OH)D) was measured by ELISA method, using 25OH Vitamin D Total ELISA Kit (DIAsource Immuno Assays, Louvain-la-Neuve—Belgium). All the determinations were done according to the manufacturers' instructions.

### Statistical Analysis

SPSS 25 was used for statistical analyses. Normal distribution for continuous variables was assessed with the Kolmogorov–Smirnov normality test. Continuous variables were expressed as mean ± standard deviation (SD) or median (Percentile 25–75), and they were compared using an unpaired Student's *t-*test or the non-parametric Mann-Whitney test. Categorical variables were presented as counts and percentages, which were compared using χ^2^-statistics or Fisher's exact test. Statistical significance was defined as *P* < 0.05.

## Results

A total of 80 children were included in the study. The patients with RTI were divided into 2 groups: those diagnosed with influenza (32 children), and those ones with a negative influenza test (27 children). The healthy control group comprised 21 children.

### Demographic Characteristics and Clinical Symptoms

The demographic characteristics and the symptoms of the study children are shown in Table 1. The age distribution of the patients was similar in all groups. Females were predominant in the group of children testing positive for influenza (53.1%), while the male patients predominated in the non-influenza and control groups (66.7%, respectively, 52.4%). With regard to the clinical symptoms, the duration of fever was similar in the influenza and non-influenza groups, but the maximum body temperature was significantly higher in the influenza group (39.79 ± 0.316 vs. 39.13 ± 0.52, *p* < 0.01), although both groups recorded a maximum temperature above 39°C. The duration of fever was lower in children with influenza compared to non-influenza group, but the difference was not statistically significant. The presence of cough was similar in both groups, around two thirds.

**Table 1:** Demographic and clinical characteristics of children attending the emergency department with respiratory symptoms and healthy control subjects.

	**Influenza group** ***N* = 32**	**Non influenza** ***N* = 27**	**Control subjects** ***N* = 21**	Statistics^*^
**Gender no (%)**				
Male	15 (46.9%)	18 (66.7%)	11 (52.4%)	
Female	17 (53.1%)	9 (33.3%)	10 (47.6%)	
Age (Median, percentiles, 95% CI)	11 (5–16) 95% CI (9.78–12.02)	10 (5–16) 95% CI (9.35–12.02)	10 (8–14) 95% CI (9.68–11.21)	*P*^1, 2^ = 0.98 *P*^1, 3^ = 0.522 *P*^2, 3^ = 0.578
Days of fever (Mean ± SD, 95% CI)	1.25 ± 0.567 95% CI (1.04–1.45)	1.59 ± 0.888 95% CI (1.24–1.94)		0.078
Fever max, (°C) (Mean ± SD, 95% CI, 95% CI)	39.79 (±0.316) 95% CI (39.67–39.90)	39.13 (±0.52) 95% CI (38.92–39.33)		<0.001
Presence of cough, number of patients (%)	20 (62.5%)	21 (77.8%)		0.262

* *P^1, 3^: influenza vs. controls, P^2, 3^: non-influenza vs. controls, P^1, 2^ influenza vs. non-influenza*.

### Laboratory Findings

The laboratory values are reported in Table 2. The WBC and the hemoglobin level (Hb) were similar in the three groups. The ESR and CRP showed no difference between the groups of children with RTI (influenza vs. non-influenza).

**Table 2:** The laboratory values of children attending the emergency department with respiratory symptoms and healthy control subjects.

**Laboratory values** **(median and range)**	**Influenza group** ***N* = 32**	**Non-influenza group** ***N* = 27**	**Control subjects** ***N* = 21**	Statistics^**^
ESR mm/h	17 (5–55) 95% CI (14.47–23.77)	15 (3–85) 95% CI (12.94–26.10)	12 (3–35) 95% CI (9.12–16.50)	*P*^1, 3^ = 0.044 *P*^2, 3^ = 0.115 *P*^1, 2^ = 0.98
CRP (mg/dl)	0.8 (0.4–1.4) 95% CI (0.74–1.5)	0.4 (0.2–1.1) 95% CI (0.47–1.6)	0.15 (0.07–0.4) 95% CI (0.13–0.4)	*P*^1, 3^ <0.001 *P*^2, 3^ = 0.01 *P*^1, 2^ = 0.964
VitD (mg/dl)	23.97 (13.88–52.69) 95% CI (21.85–28.52)	21.49 (15.47–36.2) 95% CI (20.91–24.91)	26 (16.25–79.6) 95% CI (23.95–36.77)	*P*^1, 3^ = 0.108 *P*^2, 3^ = 0.013 *P*^1, 2^ = 0.48
WBC cell/ml	5,650 (4,750–7,250) 95% CI (5,197.48–6,865.02)	5,600 (4,150–7,100) 95% CI (5,058.84–7,474.49)	6,600 (5,450–8,150) 95% CI (6,296.06–8,380.13)	*P*^1, 3^ = 0.07 *P*^2, 3^ = 0.250 *P*^1, 2^ = 0.701
PLT ×10^3^/ml	233 (161–367) 95% CI (226.37–265.51)	258 (129–438) 95% CI (238.70–296.11)	306 (174–430) 95% CI (268.67–338.95)	*P*^1, 3^ = 0.005 *P*^2, 3^ = 0.1117 *P*^1, 2^ = 0.290
PCT, %	0.20 (0.14–0.30) 95% CI (0.19–0.22)	0.21 (0.13–0.37) 95% CI (0.19–0.23)	0.23 (0.17–0.39) 95% CI (0.22–0.28)	*P*^1, 3^ = 0.011 *P*^2, 3^ = 0.025 *P*^1, 2^ = 0.951
MPV, fl	8.4 (6.5–9.9) 95% CI (8.16–8.71)	7.8 (6.5–10.5) 95% CI (7.55–8.22)	8.4 (5.9–10.7) 95% CI (7.77–8.90)	*P*^1, 3^ = 0.610 *P*^2, 3^ = 0.102 *P*^1, 2^ = 0.005
PDW %	16.65 (15.6–18.7) 95% CI (16.49–16.96)	16.3 (15.8–17.7) 95% CI (16.29–16.69)	16.3 (15.1–18) 95% CI (15.95–16.71)	*P*^1, 3^ = 0.063 *P*^2, 3^ = 0.452 *P*^1, 2^ = 0.112
MPV^*^PLT	2,020.55 (1,394–3,053) 95% CI (0.03–0.04)	2,088 (1,341.9–3,766.8) 95% CI (0.03–0.04)	2,360 (1,710–3,948) 95% CI (0.03–0.04)	*P*^1, 3^ = 0.018 *P*^2, 3^ = 0.377 *P*^1, 2^ = 0.048
VitD^*^MPV	199.46 (103.09–433.48) 95% CI (184.35–238.07)	168.26 (117.63–380.08) 95% CI (161.07–200.72)	242.48 (133.86–684.73) 95% CI (196.70–307.12)	*P*^1, 3^ = 0.102 *P*^2, 3^ = 0.004 *P*^1, 2^ = 0.048
VitD^*^PLT	5,543.29 (3,011.96–16,544.66) 95% CI (5,222.05–7,285.27)	6,128.76 (3,210.56–9,720.51) 95% CI (5,363.44–6,612.34)	7,729.05 (3,453.9–22,373.22) 95% CI (7,124.52–11,535.08)	*P*^1, 3^ = 0.006 *P*^2, 3^ = 0.003 *P*^1, 2^ = 0.840
VitD^*^PDW	398.21 (238.73–862.05) 95% CI (365.16–479.09)	355.16 (252.20–640.70) 95% CI (343.56–412.96)	433.98 (269.75–1,257.99) 95% CI (392.07–593.46)	*P*^1, 3^ = 0.102 *P*^2, 3^ = 0.010 *P*^1, 2^ = 0.315

** *P^1, 3^: influenza vs. control, P^2, 3^: non-influenza vs. control, P^1, 2^ influenza vs. non-influenza*.

*ESR, erythrocyte sedimentation rate; CRP, C-reactive protein; PLT, platelet count; PCT, Plateletcrit; MPV, mean platelet volume; PDW, platelet distribution width; Vit D, Vitamin D*.

* *denote the multiplication operation*.

**Figure 1:**
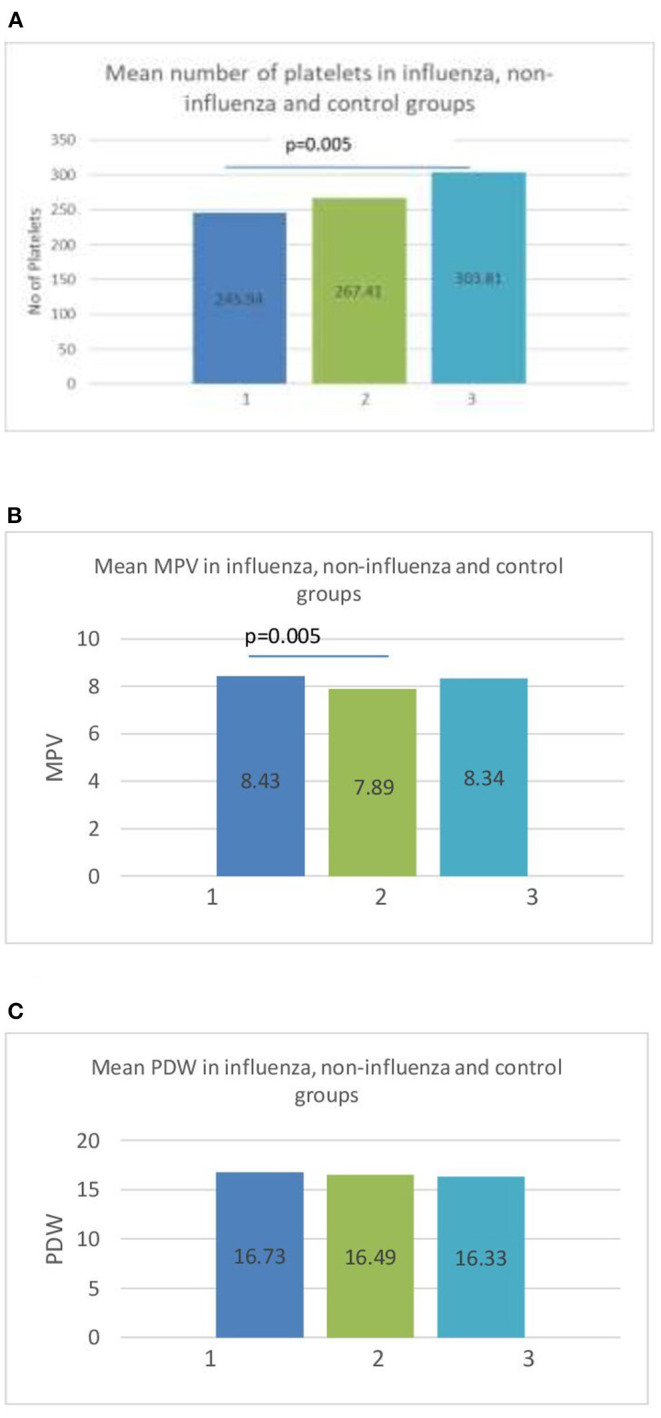
**(A)** Mean number of platelets (PLT) in influenza (1), non-influenza (2), and control (3) groups. **(B)** Mean value of mean platelet volume (MPV) in influenza (1), non-influenza (2), and control (3) groups. **(C)** Mean platelet distribution width (PDW) in influenza (1), non-influenza (2) and control (3) groups.

The platelet count (PLT) showed significant differences between groups. The children diagnosed with influenza had lower PLT (233 × 10^3^/ml vs. 306 × 10^3^/ml), although thrombocytopenia with PLT <150 × 10^3^ cells/ml was observed in only one patient, in the non-influenza group. The platelet indices were examined further among the groups. Figure 1 shows the graphic representation of mean PLT, mean platelet volume (MPV) and platelet distribution width (PDW).

**Figure 2:**
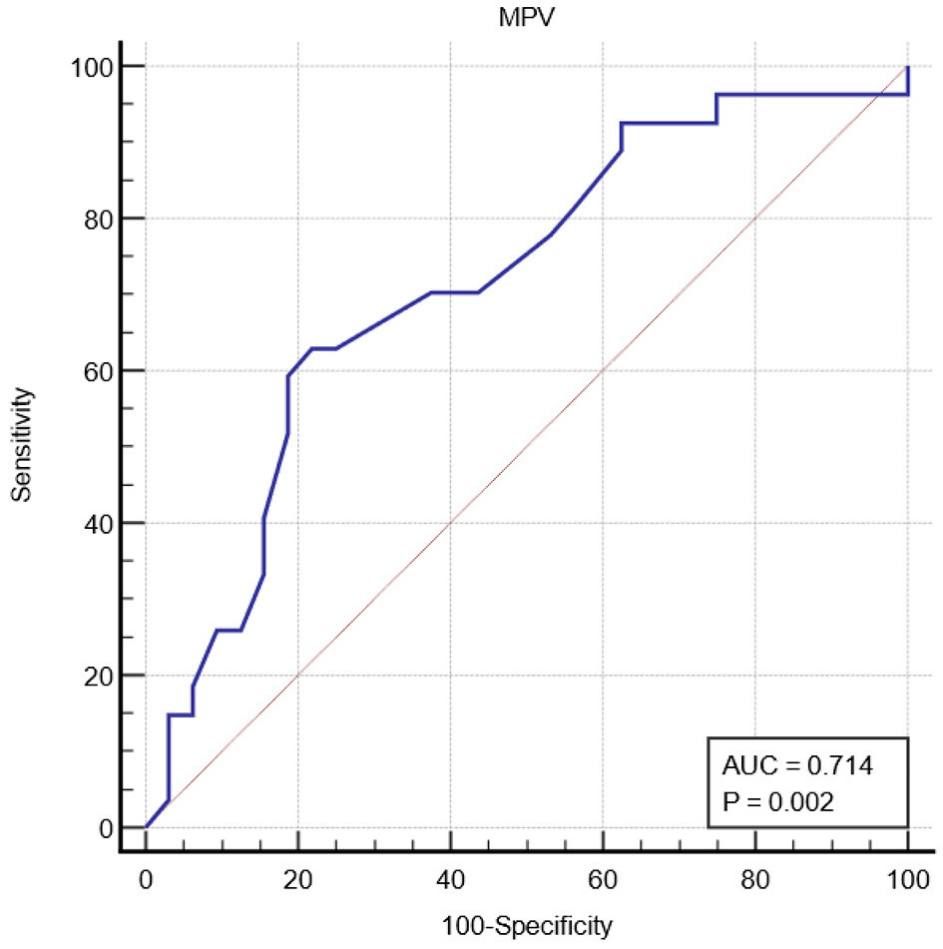
Receiver operating characteristic curve ROC of mean platelet volume (MPV) for influenza respiratory tract infection.

The MPV was significantly higher in the influenza group than in the non-influenza group (8.43 vs. 7.89, *p* = 0.005) (Figure 1B). The PDW showed no statistical difference between the groups (Figure 1C). The sensitivity and specificity of the MPV using a cut-off value of 8 fL was 62.96% [95%CI (42.4–80.6)] and 78.12% [95%CI (42.4–80.6)], respectively, in predicting influenza RTI. The ROC curve of MPV is shown in Figure 2.

The platelet mass (MPV^*^PLT) showed significant differences between the groups, being greatest in the control group and least in the influenza group: influenza vs. control [2,020.55 (1,394–3,053) vs. 2,360 (1,710–3,948), *p* = 0.018], and influenza vs. non-influenza [2,020.55 (1,394–3,053) vs. 2,088 (1,341.9–3,766.8), *p* = 0.048].

With regard to vit D levels, the non-influenza group showed significantly lower levels of vit D compared to the control subjects [21.49 (15.47–36.2) vs. 26 (16.25–79.6), *p* = 0.013]. The median level of vit D was slightly lower in the influenza group than in control group [23.97 (13.88–52.69) vs. 26 (16.25–79.6)], but the difference did not reach the level of statistical significance (*p* = 0.108). Additionally, several complex parameters resulting from the multiplication of the vit D level with MPV, PLT and PDW, respectively, are lower in the non-influence than in the control group and this difference is statistically significant.

## Discussion

The aim of this study was to evaluate the platelet response to viral RTI in children by identifying the differences in PLT and platelet indices among children suffering from influenza and other viral RTI, compared with healthy control subjects, and exploring the impact of vit D on platelet parameters. Moreover, we intended to evaluate the role of platelet indices, specifically of MPV as a potential factor to differentiate influenza by other viral RTI. The early etiological diagnosis of RTI may be difficult in certain location. The PLT indices can offer useful information and they are already determined and available through complete blood count (CBC).

None of the children with influenza developed thrombocytopenia, while only one from the non-influenza group had PLT 123 × 10^3^ cells/ml. Vit D deficiency, with vit D levels <20 mg/dl, was observed in 7/32 children in the influenza group, 7/27 in the non-influenza group and 3/21 in the control group. None of the children had very low levels of vit D of <10 mg/dl.

The two groups with RTI had similar clinical characteristics, regarding the duration of the illness, measured by the days of fever, although those in the influenza group developed higher fever, and the presence of cough. The inflammation indices CRP and ESR were similar in the two groups with RTI, and higher in both than in the control group.

The groups with RTI showed similar PLT, but the PLT in the influenza group was significantly lower than in the control group. Also, in this study, we found higher MPV in children with influenza RTI than in those with other RTI, suggesting that the PLTs are activated. MPV acted as a positive acute phase reactant which reacted differently comparing with other acute phase markers (WBC, CRP, ERS) that are not different in the two groups (*p* = 0.7, 0.96, 0.98, respectively). A higher MPV occurs as a result of increased platelet activity and thus of more intense inflammation as a result of a certain infection (27).

The mechanism of low PLT can be explained by studying further the platelet parameters, MPV and PDW. The MPV in the influenza group showed no difference from the control group, while the PDW was higher, but not to a statistically significant degree. This means that in response to influenza, the platelets maintain their size, but have increased variability, compared to the control subjects. In the non-influenza RTI group, the PLT showed no difference, but the platelet size was significantly lower compared to the control group, but with stable PDW, i.e., no significant size variability.

To explain these findings, we must review the platelet response to inflammation/infection. The platelets are cytoplasmic fragments of megakaryocytes. The MPV has been extensively evaluated, both in healthy subjects and in several medical conditions (28). The regulation of platelet production and maturity is a result of the effects of several hormones and immunological factors on the megakaryocytes. The aim is to maintain the platelet mass stable by inverse changes PLT and MPV, with the final goal being to preserve an adequate haemostatic potential (29). Thrombopoietin, as the major regulator, is positively correlated with PLT but not with MPV (30). In several pathological conditions the platelets become activated (31). This process can be related to increased production of thrombopoietin in some cases, but not in others. Various inflammatory conditions causing an increase in thrombopoiesis, with an increase in the number and the size of platelets. The platelets migrate to the area of inflammation where they are consumed, leading to thrombocytopenia. Activation of platelets is associated with increased MPV (32), which has been observed in many inflammatory conditions, including RA and Mediterranean fever (28). However, the MPV may be decreased with activity of the disease in ankylosing spondylitis RA (33), and a drop in MPV has been seen in some cases of active inflammation (34). Karadag-Oncel et al. suggested that MPV may be a useful predictor for diagnosed community-acquired pneumonia, but not in disease severity, that is to the decision for hospitalization (35).

With regard to viral infections, several different mechanisms of PLT modulation have been proposed (36), and the final outcome is increase in platelet reactivity, and activation (37). Direct interaction of viral particles with platelets has been observed, and the viruses can interact not only with circulating platelets but also with megakaryocytes. In addition, the virus-antibody complex can also activate platelets. As a result of inflammation, the platelets can migrate to, and be consumed in, the infection sites.

The defense mechanism mediated by platelets is very complex, involving activation and recruitment of leukocytes. The platelet-leucocyte activation results in vascular inflammation. Finally, platelets are also implicated in the resolution of inflammation (38).

In the case of influenza, the platelets play an important role in the host immune defense, and the inflammation process, and live viruses have been identified inside the platelets (39). It has been suggested that the initial defense against influenza is mediated by platelet-neutrophil cross-communication (40). Platelets play a major role in influenza inflammation, as it has been shown in the lungs of influenza infected mice (41). The platelet activating factor receptor plays a role in recruiting neutrophils and is associated with the final the morbidity and mortality (42). In addition, in influenza infection, the platelets are recruited by the endothelial cells at the sites of inflammation, causing lung tissue damage (43, 44). Statistical differences in PLT have been reported between influenza and COVID-19 (45).

In the current study, the children with non-influenza RTI had low MPV, but the PLT remained stable, suggesting diminished platelet activation. The platelets are not activated and consequently they are not consumed at the sites of inflammation. This may be a direct effect of the viral strains on the platelet response, or due to alterations in the innate host immunity.

With regard to the children with influenza, their mean PLT was lower than that of the control group, but with no differences in MPV. Their mean PDW was minimally elevated, but the difference did not reach statistical significance. These findings suggest platelet activation and consumption at the inflammatory sites. A dysregulation of platelet mass was observed, as PLT^*^MPV was lower than in the control subjects, due to decreased PLT.

The differences in platelet indices between the two groups with RTI, influenza and non-influenza, cannot be explained by the degree of inflammation as this was documented to be similar by the clinical symptoms and the levels of the inflammatory indices ESR and CRP. The only notable difference between the two groups was the vit D level, which was significantly lower in the non-influenza group compared to the control group.

The immunomodulatory effect of vit D has been described in several illnesses (18, 46, 47). In addition, the role of vit D has been demonstrated in respiratory diseases (48), and specifically in influenza (49). Regarding the association of PLT and other platelet parameters with vit D levels, several studies have reported the absence of any relationship under healthy conditions (50–52), but in several pathological processes the results are contradictory. Many reports have shown an inverse relationship between vit D levels and MPV. In female patients with chronic diseases, vit D levels <20 mg/dl an inverse linear relationship with MPV has been demonstrated (53). The same relationship was described at even lower vit D levels, <10 mg/dl (31, 54), in pregnant women with gestational diabetes mellitus (DM) (55) and in patients with fibromyalgia (56), although in the latter study no differences in MPV from the control subjects were noted with vit D levels 20–30 mg/dl. Our study showed that low vit D levels are associated with low MPV in children with non-influenza RTI. These results are in accordance with a study in patients with thyroid cancer, which showed low levels of MPV to be associated with lower vit D levels (57). It appears that an altered cause-effect relationship between vit D levels and MPV may develop under different circumstances. Silvagno et al. study's results suggest that the platelet activation might be modulated by a mitochondrial non-genomic activity of vit D receptor (VDR) (58). Lower vit D levels lead to a decrease in VDR expression and therefore to a decrease in PLT activation. Also, a possible mechanism could be that the lower vit D may prevent the innate inflammatory/immune response from affecting the platelet activation- destruction process. Furthermore, the deficient state of vit D in the event of viral infection may alter the virus-platelet or the inflammation-platelet interaction by affecting the metabolism of the platelets themselves. In health adults, Park et al. showed that PLT and MPV are inversely associated with vit D levels (53). Based on our findings, we consider that the interactive mechanism of vit D-platelet-inflammation/immune response is quite complex, and that the final outcome of platelet activation depends on other additional factors.

The low vit D levels could be a result of insufficient dietary intake of vit D compared to increased demand of vit D as a consequence of the infection in the non-influenza patients. It is of note that our series consists of patients who were brought to the ED with symptoms severe enough to alarm their parents. It is not clear whether the lower vit D levels led to an increase in symptom severity, resulting in the ED visit, or if the severity of the symptoms was due to the virulence of the microorganisms. In addition, the vit D status could be a cofounder to viral susceptibility.

The main strength of the paper is the demonstration of an influence of influenza virus on platelet reaction and the establishment of a direct relationship virus-platelet immune process. This study was limited to a specific age range, 4–16 years, and younger children and infants were not included. In addition, none of the children were diagnosed with severe vit D deficiency. The children were examined within the first 2 days of the beginning of illness. It would be interesting to evaluate the platelet parameters during the course of their disease. Further studies are needed to confirm our findings, and to extend the evaluation to the clinical outcome of those patients with low vit D levels and platelet inactivation.

The major limitation of this study was the small sample size of children. Our study was designed to be conducted over 2 flu seasons, 2019–2020 and 2020–2021. Unfortunately, the appearance of the pandemic, did not give us the opportunity to continue the study. Shortly after WHO declared the SARS-CoV-2 pandemic, the cases of influenza showed a sudden decrease, and the “flu season” ended earlier than usual. Next year, due to the introduction of the measures to limit SARS-CoV-2 transmission, a decrease in respiratory viral infections has been observed in children.

This study has identified alterations of platelet indices in patients with influenza and other respiratory viral illnesses in association with vit D levels. Measures of molecules secreted by activated platelets and platelets functional studies should be performed to verify the relationship of platelet parameters with the platelet activation.

## Conclusions

In conclusion, the present study showed that viral RTI in children can diminish the platelet size probably by suppressing the platelet activation. Also, we found that MPV acted as a positive acute phase reactant in children with influenza RTI, and MPV levels were significantly elevated in these children. This response is associated with low levels of vit D, which probably alters the virus-platelet-immune/inflammatory process.

## Data Availability Statement

The raw data supporting the conclusions of this article will be made available by the authors, without undue reservation.

## Ethics Statement

The studies involving human participants were reviewed and approved by Scientific Commission of General Hospital of Amaliada, Greece. Written informed consent to participate in this study was provided by the participants' legal guardian/next of kin.

## Author Contributions

GF, VV, IB, and MZ: conceptualization. GF, RP, IB, and MZ: methodology. GF, VV, and IB: analysis and writing—original draft preparation. GF, RP, and IB: investigation. GF, VV, IB, LS, and MZ: writing—review and editing. LS, AB, and MZ: supervision. GF and MZ: project administration. All authors read and agreed to the published version of the manuscript.

## Funding

This work was supported by PhD Grant 1300/24/13.01.2017, University of Medicine and Pharmacy, Iuliu Hatieganu, Cluj Napoca, Romania.

## Conflict of Interest

The authors declare that the research was conducted in the absence of any commercial or financial relationships that could be construed as a potential conflict of interest.

## Publisher's Note

All claims expressed in this article are solely those of the authors and do not necessarily represent those of their affiliated organizations, or those of the publisher, the editors and the reviewers. Any product that may be evaluated in this article, or claim that may be made by its manufacturer, is not guaranteed or endorsed by the publisher.
